# Use of osteopathic manipulation techniques for management of acute otitis media in pediatric patients: a scoping review

**DOI:** 10.1007/s00405-025-09492-9

**Published:** 2025-06-06

**Authors:** Cory Hyun-su Kim, Lauren R. McCray, Shaun A. Nguyen, Carl Shermetaro, Wayne K. Robbins

**Affiliations:** 1 Department of Otolaryngology – Head and Neck Surgery, Medical University of South Carolina, 135 Rutledge Avenue, Charleston, SC 29425-5500 USA; 2 Nova Southeastern University College of Osteopathic Medicine, Clearwater, FL USA; 3 Baylor College of Medicine, Houston, TX USA; 4 Department of Otolaryngology – Head and Neck Surgery, McLaren Health Care, Clarkston, MI USA; 5 Department of Otolaryngology – Head and Neck Surgery, OhioHealth Doctors Hospital, Columbus, OH USA

**Keywords:** Acute otitis media, Children, Pediatric patients, Osteopathic manipulative treatment (OMT), Scoping review, Ear infection, Somatic dysfunction

## Abstract

**Objective:**

To map and summarize the existing literature on the use of osteopathic manipulative techniques (OMT) in the management of acute otitis media (AOM) in pediatric patients, with an emphasis on reported outcomes and identifying gaps in the current evidence.

**Data sources:**

CINAHL, PubMed, and SCOPUS.

**Review methods:**

A comprehensive literature search was conducted across multiple databases following the PRISMA-ScR (Preferred Reporting Items for Systematic Reviews and Meta-Analyses extension for Scoping Reviews) guidelines. Studies were charted and summarized based on key characteristics, including study design, population, types of OMT applied, and reported outcomes on management of AOM and recurrent AOM in pediatric patients using OMT. No formal meta-analysis was performed, and all outcome measures were descriptively synthesized.

**Results:**

Three randomized controlled trial (RCT) studies and one pilot cohort study (*N* = 205) pertaining to application of OMT in pediatric patients with otitis media were included. Mean age for OMT and control (either sham OMT or standard of care) groups were 19.1 months and 16.8 months; proportions of males were 53.2% and 55.9%, respectively. In the pilot cohort study done by Degenhardt and Kuchera, 62.5% of the subjects experienced no documented recurrence of AOM symptoms at one year post-OMT intervention follow-up; however, since no control group was available for this study, any statistical comparison of recurrence-free rate was unfeasible. In the RCT study by Mills et al., the OMT group showed statistically significant effects on reducing frequency of mean monthly AOM episodes, resulting in fewer surgical procedures, delaying surgical interventions, increasing resolution of middle ear effusion and better tympanogram readings based on mean sum of types A and C tympanograms, and higher parental satisfaction with overall experience and perceived effectiveness of the OMT on their children on a scale of 0 to 5 when compared to the control group. While statistical interpretation showed some significance in various aspects, OMT’s clinical significance remained questionable, especially considering natural course of healing in AOM. In the other RCT study by Steele et al., at the second-week visit during the 3-week OMT intervention period, the OMT group showed a significantly higher likelihood of middle ear effusion resolution based on tympanogram findings and acoustic reflectometer measurements, respectively. However, at one month follow-up visit, there was no statistical significance, alluding to the limited effects of OMT. Finally, in the last RCT study by Whal et al., the OMT group failed to show any significant effects on prevention of recurrence of AOM.

**Conclusion:**

Current literature on the use of OMT for acute and recurrent otitis media in pediatric patients suggests, with low certainty, that OMT may provide modest benefits in reducing recurrence rates and improving middle ear function. However, the existing evidence is limited in scope and quality. Further research with larger sample sizes and rigorous randomized controlled trial designs is needed to better understand the potential role of OMT in the management of AOM in pediatric patients.

## Introduction

Osteopathic manipulative treatment (OMT) is a holistic approach to patient care that involves physical manipulation of the musculoskeletal and lymphatic systems to facilitate diagnosis and treatment of various medical conditions. This non-invasive therapy is based on the principle that the body has the inherent ability to heal itself, and OMT aims to optimize this process by addressing underlying structural and functional imbalances. Although children’s developing anatomy differs from that of adults, OMT has been found to be safe and well-tolerated in both pediatric and adult populations [1].

However, evidence regarding the effectiveness of OMT in treating various pediatric conditions has been mixed. A recently published systematic review and meta-analysis on the use of OMT for pediatric conditions concluded that while the quality of trials has improved in recent years, the effectiveness of OMT for the studied conditions remains unproven due to the overall low quality of the available evidence [1]. This underscores the need for more high-quality research to better understand the potential benefits of OMT in the pediatric setting.

Among various illnesses that affect children, acute otitis media (AOM) is the most common diagnosis during sick visits and a leading cause for antibiotic prescriptions in the pediatric population [1, 2, 3, 4]. Its incidence peaks between six and 15 months of age, and up to 85% of children will have experienced AOM by three years of age [3]. Given the substantial morbidity associated with AOM and the increasing need for antibiotic stewardship, exploring complementary treatment modalities such as OMT becomes increasingly important. Children are particularly vulnerable to AOM due to the horizontal orientation of their eustachian tubes, which can hinder proper drainage and facilitate fluid accumulation in the middle ear; additionally, somatic dysfunctions of the temporal and sphenoid bones, along with tension in the pharyngeal muscles surrounding the eustachian tube, may also contribute to the development and recurrence of AOM [5]. OMT has the potential to address these underlying anatomic factors, potentially reducing the duration and recurrence of AOM.

This scoping review aims to investigate the effectiveness of OMT on various clinical outcomes in the management of AOM and recurrent AOM in pediatric patients. The review will synthesize existing literature to evaluate the impact of OMT on symptoms, recurrence rates, and other clinical measures, ultimately aiming to identify gaps for future research in this area and assess potential role of OMT in management of AOM in pediatric patients.

## Materials and methods

This comprehensive scoping review was conducted in accordance with Preferred Reporting Items for Systematic Reviews and Meta-Analyses Extension for Scoping Reviews (PRISMA-ScR) guidelines and followed the five-stage framework outlined by Arksey and O’Malley [6, 7]. A preliminary search of PubMed, Scopus, and CINAHL was conducted, and no current scoping reviews on the topic were identified.

### Identify reseach question

This scoping review sought to investigate the potential role of OMT on AOM management in pediatric population. Furthermore, we want to highlight the clinical importance of the limitations in the current literature, as well as identify gaps in the current literature.

### Identify relevant literature

A systematic search was conducted with PubMed (US National Library of Medicine, National Institutes of Health), SCOPUS (Elsevier), and CINAHL (EBSC) on 19 July 2024, using the following keywords: osteopathic medicine; osteopathic manipulation; osteopathic manipulation technique; osteopathic manual manipulation; otitis media; ear diseases; effectiveness.

All articles from the search were exported into Covidence (Veritas Health Innovation Ltd., Melbourne, Australia), the review management software, for screening. All identified citations were collated and uploaded in EndNote X20/2021 (Clarivate Analytics, Philadelphia, PA, USA).

### Study selection

This study aimed to identify all published reports relevant to the use of OMT on treatment and prevention of AOM in children. The population of interest was pediatrics (< 16 years). This scoping review considered randomized controlled trials, non-randomized controlled trials, prospective and retrospective cohort studies, prospective and retrospective chart reviews, case–control studies, and case series studies. Review articles were assessed but not included in the reporting of quantitative data to avoid redundancy. Other exclusion criteria were adults > 16 years, study protocols, and incomplete or inaccessible articles.

#### Search process

This review was conducted in compliance with PRISMA-ScR scoping review methods [6]. The initial search yielded 341 reports with the removal of 39 duplicates. The remaining 302 titles and abstracts were screened by two independent reviewers (CHK and LRM) for assessment against the exclusion criteria, and conflicts were resolved by a third party (SAN). There were 22 full-text studies assessed for eligibility, with 18 being excluded at the full-text stage for only including adult population, wrong study design consisting of reviews and editorial letter, and full text being unavailable. Four full-text screenings were completed independently by the same reviewers (CHK and LRM) and any disagreements were resolved through discussion. The results of the study inclusion process are presented in a PRISMA-ScR flow diagram.

### Charting the data

#### Data extraction and level of evidence

Data extraction from the four reports included was performed by two independent reviewers (CHK and LRM) and any disagreements were resolved through discussion. The data extracted from reports included author; year of publication; study design; patient demographics, such as age and gender; recurrence of AOM; frequency of episodes of AOM; antibiotic use; surgical interventions; tympanometric, audiometric, and acoustic reflectometer readings; and resolution of somatic dysfunctions in entire body, including thoracic, rib, and cranial regions. When available, other relevant data, such as quality-of-life measures, duration of otorrhea, duration of otalgia and fever, language skills, and parental satisfaction of overall care were also extracted. In addition, all included reports were critically appraised to assess the level of evidence using the Oxford Center for Evidence-Based Medicine criteria [8].

### Collating, summarizing and reporting results

Review results are presented through descriptive statistics (frequency (%), mean/median, and range/95% confidence interval (CI)) of included studies and a narrative summary of findings. Implications of the analysis are then discussed.

## Results

### Publication characteristics

**Fig. 1:**
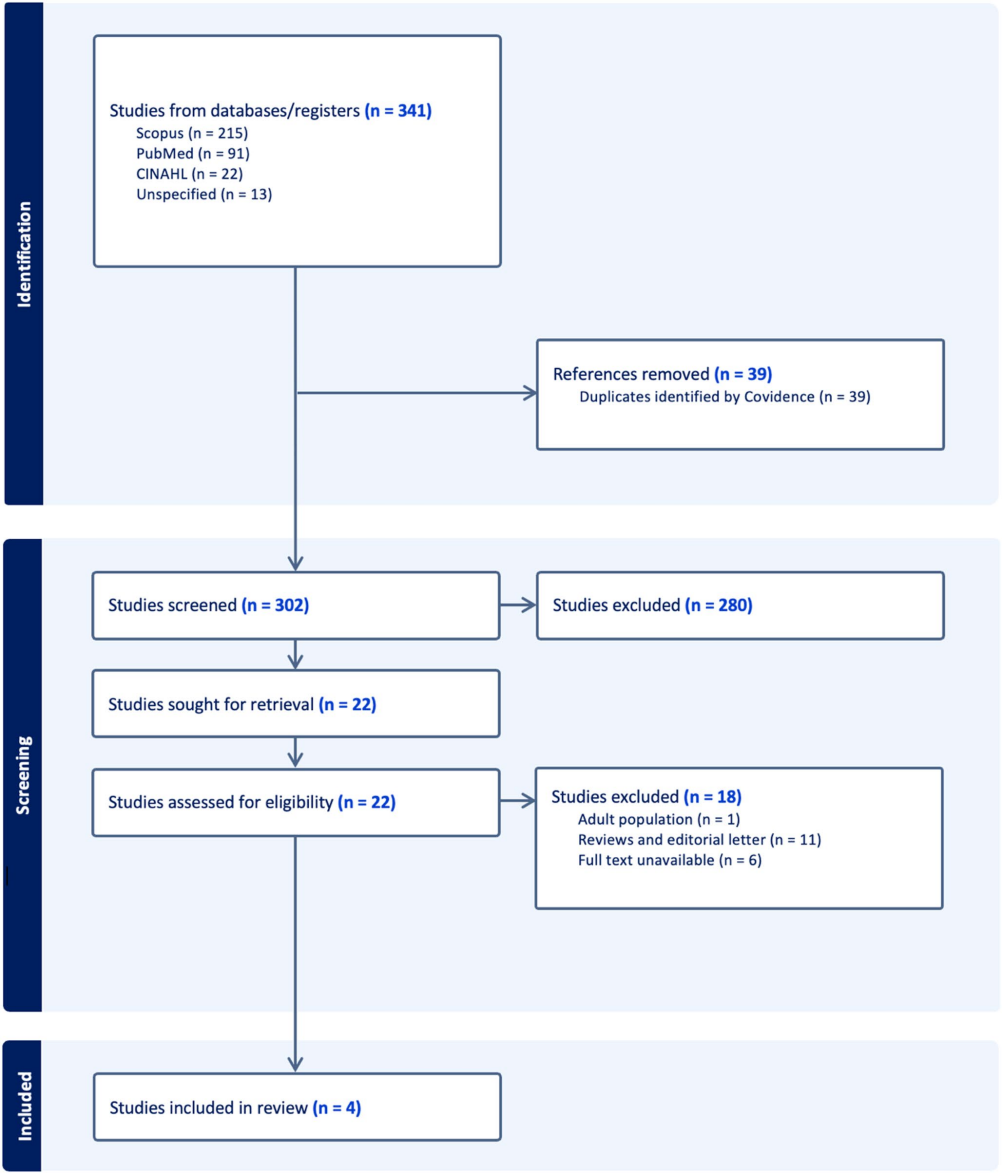
Preferred Reporting Items for Systematic Reviews and Meta-Analyses (‘PRISMA’) diagram

The four studies included were one pilot cohort study (*N* = 8) and three randomized control trials (RCTs) (*N* = 197) published between 2003–2014 [9, 10, 11, 12]. A PRISMA diagram outlining our search is provided in Fig. 1. Descriptions of the individual studies and selected patient characteristics can be seen in Table 1.

**Table 1:** Summary of study characteristics including authors, Oxford level of evidence, number of patients, intervention on study and control groups, OMT used in the study, and main outcome measures

Study	Oxford Level of Evidence	Patients (*n*)	Intervention on Control and Study Groups	OMT Used in the Study	Outcomes
Degenhardt and Kuchera (2006) [9]	3	8	Weekly OMT with concurrent traditional medical management for 3 weeks	Gentle balanced membranous tension (cranial) and myofascial release for somatic dysfunctions of entire body	Recurrence of AOM since OMT intervention at 1-year post-treatment follow-up
Mills et al. (2003) [10]	2	57	Control: routine pediatric care Study: routine pediatric care plus OMT	Gentle techniques on areas of restriction consisting of articulation, myofascial release, balanced membranous/ligamentous tension, facilitated positional release, and/or counter-strain treatments on entire body. No high-velocity or thrusting maneuvers were used.	Frequency of episodes of AOM, antibiotic use, surgical interventions, various behaviors, and tympanometric and audiometric performance
Steele et al. (2014) [11]	2	43	Control: standard care only (SCO) Study: standard care (SC) plus OMT during 3 weekly visits	Balanced ligamentous tension and myofascial release on pelvis, thoracolumbar junction, rib cage, diaphragm, and cervical area; suboccipital inhibition, venous sinus drainage technique, occipital decompression technique, and sphenobasilar symphysis decompression technique	Weekly tympanometric and acoustic reflectometer readings
Wahl et al. (2008) [12]	2	84	Four protocol groups: (1) double placebo (2) echinacea plus sham OMT (3) true OMT plus placebo echinacea (4) true echinacea plus OMT	Cranial osteopathy, balanced membranous/ligamentous tension, and/or myofascial release on the cranium, pelvis, diaphragm, and other structures. No high velocity or thrusting maneuvers were performed. Osteopathic percussion hammer was also used at the discretion of the osteopathic physician.	Occurrence of a first episode of AOM during the study period and number of episodes of AOM

### Patient attributes

The studies include pediatric patients with acute or recurrent otitis media. All four papers considered in this review excluded subjects that have chromosomal abnormalities, major congenital malformations of the head and neck, immunologic abnormalities or deficiencies. There are 205 total patients with 105 who received OMT and 100 who had standard of care treatment or sham OMT. The average age of the experimental group was 19.1 months, and the average age of the control group was 16.8 months (Δ = 2.3 months, 95% CI: 1.08 to 3.52 months; *p* = 0.0003); the total age range was 6 months to 6 years. The experimental group was 53.2% male, while the control group was 55.9% male (Δ = 2.7%, 95% CI: -10.76 to 16.01%; *p* = 0.70). The number of patients who attended daycare was 56.9% in the experimental group compared to 52.9% in the control group (Δ = 4.0%, 95% CI: -9.47 to 17.28%; *p* = 0.57), and the number of patients with smoke exposure in the home was 13.2% in the experimental group and 20.0% in the control group (Δ = 6.8%, 95% CI: -3.45 to 17.09%; *p* = 0.19).

### Outcomes

#### Degenhardt and Kuchera

This pilot cohort study included eight patients ranging from 7 to 35 months old with a history of recurrent AOM who received OMT concurrent with traditional medical management for three weeks, and they were reassessed for somatic dysfunction and posttreatment recurrence of AOM at 1-year follow up by telephone interviews with the subjects’ parents or legal guardians and chart review of patient records provided by their primary care physicians. This study had no drop-out subjects. However, due to the lack of participants, this study did not include a control group and opted to serve as a pilot cohort study exploring the initial findings and gathering preliminary data on the efficacy of OMT in pediatric AOM management. Both osteopathic examination and OMT were conducted by the two primary investigators who are osteopathic physicians.

Five (62.5%) subjects experienced no documented recurrence of AOM symptoms at one year post-OMT intervention follow-up. Notably, one subject who had a history of monthly ear problems and three prior episodes of AOM and another subject who had 12 AOM episodes in the previous year had no recurrence post-OMT treatment. Other subjects had varying outcomes, with some experiencing future episodes, including one subject who underwent surgery after recurrence at 6 weeks post-OMT treatment. In addition, many subjects experienced resolution of various forms of somatic dysfunction such as sphenobasilar synchondrosis in the base of head, ribs, pelvis, and sacrum throughout their OMT treatment period. The study authors suggest that the resolution of somatic dysfunction could have contributed to the positive outcomes observed in AOM management in pediatric patients. No otoscopic or audiometric outcome measures were reported in this study.

#### Mills et al.

Fifty-seven patients ranging from 6 months to 6 years old with recurrent AOM were randomized to receive either routine pediatric care or OMT plus routine pediatric care for 21 weeks. Outcome measures included recurrence of AOM, need for antibiotics or surgical intervention, behavior rating scales, and tympanometric and audiometric data. The study initially enrolled 76 patients, but 19 subjects—approximately 25%—dropped out due to loss of continuity of physician care and the inconvenience of the 6-month study duration. OMT was administered by four osteopathic physicians located in different sites. The study aimed to standardize the approach to OMT by utilizing only physicians with teaching experience in OMT.

The OMT group had a lower mean monthly number of AOM episodes than the control group (0.19 vs. 0.27), which was a significant difference (Δ=-0.14, 95% CI: -0.27 to 0.00; *p* = 0.04). The OMT group also had fewer mean monthly antibiotic prescriptions compared to the control group (0.30 vs. 0.42), although this difference was not statistically significant (Δ =-0.17, 95% CI: -0.38 to 0.05; *p* = 0.13). Moreover, children in the OMT group remained surgery-free for an average of 6 months, while the control group averaged 5.25 months (Δ = 0.75, 95% CI: 0.16–1.34; *p* = 0.01). One patient in the intervention group (4%) and 8 patients in the control group (25%) underwent surgical intervention (*p* = 0.03), involving only insertion of ventilatory tubes in each case; tube insertion was done at 6 month after randomization in the intervention patient (*n* = 1) and at 2 months (*n* = 2), 3 months (*n* = 4), and 4 and 6 months (*n* = 1 each) in the control group. There was no significant difference in behavior outcomes or audiometric data, although both groups showed improvement in their audiograms. However, there was a significantly higher mean sum of types A and C tympanograms in the OMT group compared to the control (1.41 vs. 1.00; Δ = 0.55, 95% CI: 0.08 to 1.02; *p* = 0.02), suggesting better middle ear function. Type A, B, C, and O tympanograms were obtained at the monthly visits and scored 0, 1, or 2; due to the small sample size, types A and C tympanograms were combined because they indicated some movement of the tympanic membrane, whereas types B and O tympanograms signify greater abnormalities such as middle ear effusion and perforated tympanic membrane. By focusing on types A and C, the authors emphasize cases where there is either normal or partially compromised function, which is more relevant for understanding whether and how the OMT influences middle ear health. The study does not provide detailed tympanometric data, such as the proportion of children presenting with a type B tympanogram at the conclusion of the study. Lastly, when asked to rank satisfaction regarding overall experience and perceived effectiveness of the OMT on their children who were part of the study on a scale of 0 to 5, with 5 indicating very satisfied, parents from the OMT group reported significantly higher scores compared to the control group (4.84 vs. 4.50; Δ = 0.34, 95% CI: 0.05 to 0.63; *p* = 0.02).

#### Steele et al.

Forty-three patients ranging from 6 months to 2 years old with AOM were randomized to receive either standard care only (SCO) or standard care plus OMT (SC + OMT) for three weeks following the baseline visit (Visit 1). Out of 52 subjects who were initially enrolled, 9 dropped out, resulting in a dropout rate of 17.3%. A total of 7 osteopathic physicians administered OMT, including two authors of the study, at two different sites.

Patients were monitored with weekly tympanometric and acoustic reflectometer (AR) readings. Tympanogram analysis at two weeks post-baseline (Visit 2) showed a statistically significant improvement in the resolution of middle ear effusion (MEE) in the SC + OMT group compared to the SCO group; the odds ratio for resolution in the SC + OMT group was 2.98 (95% CI: 1.16 to 7.62; X^2^ test for independence; *p* = 0.02), indicating a higher likelihood of resolution and better middle ear function compared to the SCO group. The number of ears that resolved to normal tympanogram readings was higher in the SC + OMT group compared to the SCO group (26 vs.16), as was the resolution rate (68.4% vs. 42.1%). In addition, the SC + OMT group showed a statistically significant improvement in AR readings at Visit 2 compared to the SCO group, with an odds ratio of 2.73 (*p* = 0.04), meaning that children receiving OMT were more likely to have resolution of MEE. However, at one month post-baseline (Visit 3), there was no statistically significant difference in AR readings between the two groups, with an odds ratio of 0.94 (*p* = 0.54). There were also no significant differences in parent responses regarding their children’s condition, indicating similar perceptions of treatment effectiveness between groups.

#### Wahl et al.

Eighty-four children aged 12–60 months with recurrent AOM were randomized to receive either double placebo, echinacea plus sham OMT, true OMT plus placebo echinacea, or true echinacea plus OMT for prevention of recurrence of AOM; they were followed for at least three months for recurrence of AOM. Out of the 90 children initially enrolled in the study, 6 subjects—approximately 7%—withdrew or were lost to follow-up within 3 months of enrollment. In terms of attendance for scheduled visits, only 19% of subjects attended all five scheduled osteopathic visits, though 64% managed to attend three or more visits. OMT was administered by osteopathic physicians of the Tucson osteopathic community with practices restricted to manipulative treatment.

The overall AOM recurrence rate was 52%, and the cumulative incidence of AOM varied significantly (*p* = 0.04) among treatment groups, with recurrence rates of 39%, 44%, 61%, and 80% for the OMT plus placebo, double placebos, true echinacea plus OMT, and echinacea plus sham OMT groups, respectively. However, although the adjusted relative risk and Kaplan-Meier estimates showed a trend towards reducing AOM with OMT, there was no statistically significant effect. Parents of children assigned to the OMT group were significantly more likely to believe that their child was receiving genuine OMT compared to parents of children in the sham treatment group. Interestingly, by the six-month follow-up, the ability of parents to distinguish between OMT and sham treatment diminished significantly, suggesting parents may have become less certain about the nature of the treatment their child was receiving and the preventative effects of OMT. Overall, the study remains inconclusive on the potential role of OMT in prevention of recurrent AOM as many of its findings were not statistically significant.

## Discussion

There is an inconclusive role and benefit of OMT for managing AOM in pediatric patients. In their study, Degenhardt and Kuchera suggest that use of OMT could significantly reduce recurrence of AOM in pediatric patients, citing that 62.5% of their subjects had no documented recurrence. This was compared to recurrence rates reported in a separate study of another population, in which only 22–38% of patients had no recurrence [13]. However, this comparison should be interpreted with scrutiny and limitation, as it lacks a proper control group and direct comparative design. Mill et al.. showed statistically significant effects of OMT in lowering mean monthly number of AOM episodes, fewer surgical procedures, delaying surgical intervention, improving middle ear function based on types A and C tympanograms scores, and garnering higher parental satisfaction on overall experience and effectiveness of the OMT on their children. While delaying surgical intervention may reflect the reassuring effects of OMT in treating AOM in pediatric patients, it could also be perceived as deferring definitive treatment that some children may ultimately require, especially given the inconclusive evidence supporting OMT’s effectiveness in pediatric AOM management. Moreover, although Mill et al.. reported a statistically significant delay in surgical intervention with OMT, the actual mean difference of just three weeks may be of limited clinical relevance. Additionally, the statistical difference in mean monthly number of AOM episodes between the OMT and control group was 0.86. This suggests that children in the OMT group experienced approximately one fewer episode of AOM per month on average. However, considering this reduction occurred after 21 weeks of OMT intervention, the clinical significance of this finding may be limited. The study by Steele et al. demonstrated statistically significant improvement in resolution middle ear effusion with OMT, as evidenced by higher resolution rate, tympanogram findings, and acoustic reflectometer readings at Visit 2. However, the absence of a significant difference in acoustic reflectometer readings between the OMT and control groups at Visit 3, which was after one month of initial OMT intervention session, suggests that the observed improvement may, in part, reflect the natural resolution course of AOM. Nevertheless, these findings may still support the argument that OMT has a potential role in accelerating the resolution process. Finally, Wahl et al.. study did not show any statistically significant benefit in the management of pediatric AOM. In fact, the recurrence rates of AOM were 39% in the OMT plus placebo group and 44% in the double placebo group, further highlighting the likelihood of natural resolution course of AOM in this population.

While the American Academy of Pediatrics (AAP) recommends a nuanced approach in treating pediatric AOM based on the child’s age, severity of symptoms, and whether the AOM is unilateral or bilateral, for children aged 6 months to 2 years with severe symptoms or bilateral AOM, antibiotics are typically recommended [14, 15–16]. However, growing concerns over antibiotic resistance and potential adverse effects have emphasized the need for complementary and alternative treatment options, such as OMT [17]. Oher treatments for AOM, particularly for recurrent AOM with unilateral or bilateral middle ear effusion, include surgery such as tympanostomy tube placement, which could significantly add to overall treatment burden and healthcare costs [18, 15]. Considering reduction of such undesired outcomes, the findings reported by Mills et al. are favorable. They found a lower mean monthly number of AOM episodes in the OMT group compared to the control, fewer monthly antibiotic prescriptions, as well as fewer surgical procedures and longer periods without the need for surgery. However, as mentioned before, delaying surgical intervention can be perceived as deferring definitive treatment that is needed. Future studies could clarify the role of OMT in preventing the need for surgery—rather than merely delaying it—by conducting controlled trials in pediatric patients with AOM who are surgical candidates and evaluating whether OMT reduces the proportion of patients who ultimately require surgical intervention.

Another management strategy that is being adopted as routine care for non-severe AOM is ‘watchful waiting’, a deferment of antibiotics and close monitoring of otitis media symptom progression [19]. Its endorsement is based on high rates of spontaneous resolution of otitis media in pediatric patients and the advantage of avoiding adverse effects of antibiotics [19, 20]. The current clinical practice guideline of AOM by the AAP recommends initial observation with close follow-up for AOM in children 24 months or older without severe signs or symptoms [15, 16]. For otitis media with effusion (OME), American Academy of Otolaryngology – Head and Neck Surgery (AAO-HNS) also recommends clinicians to manage the child with OME who is not at risk with watchful waiting for 3 months from the date of effusion onset or 3 months from the date of diagnosis [15]. During the period of ‘watchful waiting’, OMT can be incorporated to facilitate the healing process as a minimally invasive and drug-free complementary medicine. An important future research opportunity involves assessing the role of OMT during the ‘watchful waiting’ period in pediatric patients with AOM. A randomized controlled trial could evaluate whether OMT with ‘watchful waiting’ accelerates symptom resolution compared to ‘watchful waiting’ alone in this patient population.

In otitis media management, there are various other complementary and alternative medicine (CAM) one can explore besides OMT such as acupuncture, homeopathy including herbal medicine, xylitol, vitamin D supplements, and probiotics. While anecdotes of success with these modalities are present, a lack of double-blind, randomized, and controlled studies makes it hard for physicians to consider them as potential treatment of otitis media [17–21]. This holds also true for OMT based on the current literature, as more rigorous research is needed in this area. However, one noteworthy advantage that OMT may provide compared to the other options is that OMT is generally performed by osteopathic physicians, at least in the United States, conferring a holistic level of medical oversight and expertise in diagnosis and treatment.

Based on our scoping review, the studies on OMT for management of pediatric AOM are limited in multiple aspects. First, many studies had limited sample sizes, with participant numbers ranging from as few as 8 to a maximum of 84, which could reduce the statistical power and hinder the detection of significant differences between groups. Additionally, two out of five reviewed studies had high dropout rates of up to 25% and incomplete compliance with scheduled OMT visits, where only 19% of subjects attended all five planned visits. This may have diluted the potential effects of OMT, making it difficult to accurately assess its efficacy. The absence of a control or sham treatment group in some studies further raises concerns about the validity of the results, as placebo effects cannot be ruled out. Without a comparison group, it is challenging to determine whether any observed improvements were due to the OMT intervention or other factors, such as spontaneous recovery or a placebo response. The variability in treatment sessions is another limitation, compounded by the fact that OMT is highly dependent on the practitioner’s level of experience and expertise in techniques, leading to inconsistencies in treatment administration across the studies. This variability in the intervention makes it difficult to draw clear conclusions about the efficacy of OMT. A standardized protocol for OMT is for management of pediatric AOM is needed for future studies. However, given that each patient may present with unique somatic dysfunctions that OMT is intended to address, developing a single, universally applicable protocol may pose practical challenges. Other notable limitations include short observation periods, challenges with follow-up, and lack of blinding for participants and assessors. In addition, reliance on subjective measures such as parental satisfaction may introduce reporting bias, as parents’ assessments may be influenced by their personal experiences and beliefs rather than objective clinical outcomes.

Further research is warranted to address the current gaps in the literature and draw a universal conclusion on the potential role of OMT in management of pediatric AOM. First, future studies should include larger and more diverse participant groups to enhance the statistical power and generalizability of findings. Secondly, improved study design such as randomized controlled trials including a control group or a sham group in future research is essential to accurately assess the true efficacy of OMT. In addition, developing and adhering to standardized OMT protocols across studies would minimize variability in treatment application and allow for more reliable and consistent results. Ensuring a high level of expertise in OMT administration by each practitioner is crucial, given that the effectiveness of OMT is highly dependent on the skill and experience of the practitioner. Extending the follow-up duration in future studies would provide valuable information on the long-term effects and sustainability of OMT benefits. This is particularly important for chronic conditions like recurrent otitis media, where long-term outcomes are critical. Moreover, future studies should try to implement double-blinding procedures, where both participants and assessors are unaware of the treatment group assignments to reduce bias. Lastly, using more objective outcome measures, rather than subjective outcome measures such as parental satisfaction, whenever possible would reduce the influence of subjective reporting and potential parental bias. The use of validated quality of life surveys in appropriate pediatric patients and parents of the participating children would also be beneficial in assessing treatment outcomes.

## Conclusion

Acute otitis media (AOM) is among the most prevalent illnesses affecting pediatric populations. Osteopathic manipulative treatment (OMT) is a form of complementary and alternative medicine used to diagnose and treat various conditions through physical manipulation of the somatic structures.

Overall, the studies reviewed reveal with low certainty that OMT may offer some small effects in reducing recurrence rates and improving middle ear function for AOM in pediatric patients. Although some statistically significant results are reported, their clinical relevance needs further evaluation. The variability in outcomes across studies, along with the challenges in distinguishing OMT effects from placebo, indicates that further research is needed. Future studies should focus on larger, blinded, randomized controlled trials with standardized OMT protocol and well-defined outcome measures to better understand the role of OMT in pediatric AOM management and to provide clearer guidance for clinicians considering its use in practice.
